# Pharmacological activation of the nuclear receptor REV-ERB reverses cognitive deficits and reduces amyloid-β burden in a mouse model of Alzheimer’s disease

**DOI:** 10.1371/journal.pone.0215004

**Published:** 2019-04-11

**Authors:** Deborah A. Roby, Fernanda Ruiz, Bailey A. Kermath, Jaymie R. Voorhees, Michael Niehoff, Jinsong Zhang, John E. Morley, Erik S. Musiek, Susan A. Farr, Thomas P. Burris

**Affiliations:** 1 Department of Pharmacology and Physiology, Saint Louis University School of Medicine, St. Louis, MO, United States of America; 2 Center for Clinical Pharmacology, Washington University School of Medicine and St. Louis College of Pharmacy, St. Louis, MO, United States of America; 3 Department of Internal Medicine, Saint Louis University School of Medicine, St. Louis, MO, United States of America; 4 Department of Neurology, Washington University School of Medicine, St. Louis, MO, United States of America; Nathan S Kline Institute, UNITED STATES

## Abstract

Alzheimer’s disease currently lacks treatment options that effectively reverse the biological/anatomical pathology and cognitive deficits associated with the disease. Loss of function of the nuclear receptor REV-ERB is associated with reduced cognitive function in mouse models. The effect of enhanced REV-ERB activity on cognitive function has not been examined. In this study, we tested the hypothesis that enhanced REV-ERB function may enhance cognitive function in a model of Alzheimer’s disease. We utilized the REV-ERB agonist SR9009 to pharmacologically activate the activity of REV-ERB in the SAMP8 mouse model of Alzheimer’s disease. SR9009 reversed cognitive dysfunction of an aged SAMP8 mouse in several behavioral assays including novel object recognition, T-maze foot shock avoidance, and lever press operant conditioning task assessments. SR9009 treatment reduced amyloid-β 1–40 and 1–42 levels in the cortex, which is consistent with improved cognitive function. Furthermore, SR9009 treatment led to increased hippocampal PSD-95, cortical synaptophysin expression and the number of synapses suggesting improvement in synaptic function. We conclude that REV-ERB is a potential target for treatment of Alzheimer’s disease.

## Introduction

REV-ERBs (REV-ERBα and β) are nuclear receptors that function as a ligand-dependent suppressors of gene transcription and are a critical components of the mammalian circadian clock [1–5]. Additionally, the REV-ERBs play important roles in physiological pathways including metabolism and inflammation [6–8]. Although their role in circadian behavior has been known for some time, more recent studies suggest that they also play an important role in learning and memory. *Rev-erbα* null mice display impaired short-term, long-term, and contextual memory and increased hippocampal neurogenesis [9–11]. REV-ERB recruits corepressors and class 1 histone deacetylases (HDACs) to target gene promoters and class 1 HDACs have also been implicated in learning and memory [12]. A number of synthetic agonists that activate REV-ERBs’ transcriptional suppressor activity have been designed to assess the effects of modulating REV-ERB activity in vivo [13,14], but no studies examining their effects on cognitive function have been described.

Impaired cognitive function is a hallmark of Alzheimer’s disease (AD) [15,16] and is characterized by accumulation of senile plaques composed of insoluble Amyloid-β (Aβ) peptides [17], neurofibrillary tangles [18], synaptic failure [19,20], neuroinflammation [21], and mitochondrial dysfunction [22]. Interestingly, disruption of normal sleep cycles and the circadian rhythm is also closely associated with AD [23], suggesting manipulation of these pathways may hold utility in treatment or prevention of the disease. A number of animal models have been developed for studying AD pathogenesis as well as in evaluation of novel therapies, which include very commonly utilized genetically modified mice that carry mutations in genes identified in patients with familial AD [24]. However, the majority of cases of AD are polygenic, or sporadic in nature [25]. The senescence accelerated mouse prone 8 (SAMP8) is a model of sporadic AD where the mice display age-related severe cognitive deficits by 12 months of age [26–28]. They develop pathological features of AD such as impaired Aβ efflux from the brain [29,30], hyper-phosphorylated tau [31], elevated amyloid precursor protein [32,33], markers of synaptic dysfunction [34], and key changes in hippocampal gene expression [35].

Given that genetic REV-ERBα loss of function results in cognitive impairment [9], we sought to determine if pharmacological REV-ERB gain of function may improve cognitive function in a model of cognitive impairment and in particular AD. In the current study, we used the SAMP8 AD mouse model to evaluate whether a REV-ERB agonist (SR9009) could alter the time-dependent cognitive decline observed in these mice. We determined that SR9009 reverses cognitive deficits in the SAMP8 model in multiple cognitive assays by reducing Aβ levels and rescuing glial and neuronal health.

## Materials and methods

### Animals and treatment

Experimentally naïve male SAMP8 mice (virus-free in-house colony) ages 4 months and 12 months were held in a temperature-controlled facility under 12–12 light and dark conditions (light on at 6 AM). Food and water were provided ad libitum. This study was carried out in accordance with the recommendations in the Guide for the Care and Use of Laboratory Animals of the National Institutes of Health. Animal care and experimental protocols used in this study were approved by the Saint Louis University Institutional Animal Care and Use Committee (Assurance number: 2532). SAMP8 animals were divided into 3 groups: 4-month-old mice receiving vehicle (Y-V), 12-month-old mice receiving vehicle (O-V), and 12-month-old mice receiving SR9009 (O-SR9009). The Rev-Erb agonist SR9009 was administered intraperitoneally once daily at Zeitgeber time (ZT) 0 at 100 mg/kg mouse body weight for 14 days before behavior testing commenced. Vehicle was 6% v/v DMSO and 10% v/v cremophor in water at a pH of 7. Total mice used was 72.

### Novel object recognition

All behavioral assays for SAMP8 were conducted beginning two weeks post-onset of dosing. Learning and memory deficits were assayed using novel object recognition (NOR) as previously described [31]. SAMP8 mice were habituated to the maze on day 15, then exposed to two identical objects on day 16 (acquisition). Twenty-four hours later on day 17, the animals were exposed to one familiar object and one novel object (retention). Time spent with novel object was calculated as a discrimination index. Group size was 11–17. Mice were tested between ZT2-ZT6. Time spent with novel object was calculated as a discrimination index (DI), defined as (T_new_−T_old_) / (T_new_ + T_old_), as previously [36].

### T-maze foot-shock avoidance

T-maze assay was conducted as previously described [37–39]. Briefly, animals were not permitted to explore the maze prior to testing. Animals were placed in the apparatus, and simultaneously a buzzer sounded while the start box door was raised. A constant, 0.35 mA foot shock began after 5 seconds. Mice avoided the shock by entering a clear escape box in one of the arms of the T-maze, and mice were trained to number of trials necessary to learn avoidance of the foot shock (acquisition). After one week, the number of trials required for the animals to make 5 avoidances out of 6 trials (retention) were recorded. Group size was 9–12. Mice were tested between ZT2-ZT6.

### Lever press

Animals were habituated to evaporated milk with sucrose overnight for 3 days prior to testing. On the final night of habituation consumed volume was measured. Animals were separated into two groups and tested every other day for a total of 4 testing days. Animals were fasted overnight and placed in clear lever press boxes for 40 minutes per animal. Lever presses and reward retrievals were measured using the Coulbourn Instruments LabLinc system. Data was analyzed on Graphic State 3.02 software. Animals were trained for 1 day, wherein if the animals pressed the lever, the reward delivery arm was raised for 11 seconds. The subsequent 4 days, trial days, when the lever was pressed, the reward was available for 6 seconds. The number of each rewarded lever press was recorded (reward retrieval). Group size was 9–12. Mice were tested between ZT2-ZT6.

### Amyloid-β analysis

Soluble Aβ was analyzed using ELISA. ELISA services provided by Confluence Discovery Technologies in St. Louis, MO. Kit was V-PLEX Aß Peptide Panel 1 (4G8) for human, mouse, and rat (#Cat: K15199E-1) from Meso Scale Diagnostics, LLC. Cortex tissue was homogenized in 500μL reassembly buffer (50 mM Tris-HCl, pH 7.4, 150 mM NaCl, Pierce protease and phosphatase inhibitor mini tablets [Thermo Fisher], 20 mM NaF, 1 mM Na3VO4, 0.5 mM MgSO4). Samples were incubated on ice for 30 min, then centrifuged at 150,000 g for 20 min at 4°C. Supernatants were then transferred to 50 μl 0.5% Tween-20 in reassembly buffer, mixed and stored in 105μL aliquots as reassembly buffer-soluble fraction. Group size was 5.

### Immunoblotting

Brain tissue was rapidly dissected out from hippocampus and temporal cortex and flash frozen in liquid nitrogen. Tissue samples were homogenized in RIPA buffer with protease and phosphatase inhibitors (Roche). Samples were centrifuged, and protein was collected from supernatant. Primary antibodies were PSD-95 (ThermoFisher, MA1-045) at 1:1000, synaptophysin (Abcam, ab32127) at 1:1000, beta-actin (mAbcam 8226) at 1:5000. Secondary antibodies were HRP-tagged anti-mouse and anti-rabbit (Santa Cruz) 1:5000. Group size was 3.

### Histology

SAMP8 animals were anesthetized with 10 mg/mL ketamine HCl and 1 mg/mL xylazine at a dosage of 0.1 mL/10g mouse body weight injected i.p. The thoracic cavity was opened, and animals were perfused transcardially with 20 mL of phosphate buffered saline (PBS) and heparin. The brains were subsequently removed, halved, and the left hemisphere fixed in 4% paraformaldehyde, then in 30% sucrose to aid in sectioning. Brains were then serially sectioned at 50 μm on a microtome with tris buffered saline (TBS) and sucrose and preserved in 30% ethylene glycol cryoprotectant. Sections were then processed free-floating in Netwells for GFAP (Dako) at 1:500 and ZO-1 (Thermo-Fisher) at 1:250 for immunofluorescence. Alexa Fluor 488 goat anti-rat and Alexa Fluor 568 goat anti-rabbit were used at 1:5000. 2 sections per mouse were analyzed. Co-staining quantification and measurement of blood vessel diameter were performed with ImageJ. Sections were viewed on either an Olympus BX41 EpiFluor or an Olympus FV1000 Confocal microscope. Group size was 5.

### Transmission electron microscopy

Tissue was prepared as described [40]. Briefly, SAMP8 animals were anesthetized with 10 mg/mL ketamine HCl and 1 mg/mL xylazine at a dosage of 0.1 mL/10g mouse body weight. They were then perfused transcardially with PBS and 2.5% glutaraldehyde. Tissue sections were removed from the left hemisphere of the brain and pieces no more than 2mm^3^ were cut to contain both hippocampus and cortex. Tissue was then prepared according to protocol and viewed in a JEOL 1400 Plus electron microscope equipped with an AMT digital camera. 4 sections per group were analyzed for axon health. Sections were also imaged in cortex and total number of synapses were counted in each image. Group size was 4.

### Statistical analysis

Statistical analysis for tests containing three test groups was conducted using 1-way ANOVA or 2-way ANOVA. We used Newman-Keuls multiple comparisons test to identify significantly different means.

## Results and discussion

Since genetic *Rev-erbα* loss of function results in reduced cognitive function, we hypothesized that increased REV-ERB activity may increase cognitive function in diseases where cognitive function is impaired. We used the SAMP8 mouse model of premature aging and AD to assess the ability of a REV-ERB agonist to alter cognitive function. We treated one set of “old” 12-month-old SAMP8 mice with SR9009 (O-SR9009; 14 days, 100 mg/kg q.d.), one set of 12-month-old SAMP8 mice with vehicle (O-V), and one set of “young” 4-month-old SAMP8 mice with vehicle (Y-V) and subjected them to three different behavioral tasks: novel object recognition (NOR), T-maze foot shock avoidance, and lever press operant conditioning. In the NOR test, reduced time spent exploring a novel object (reduced discrimination index) is associated with reduced cognitive function (reduction in the natural tendency of mice to investigate novel aspects of their environment). The hippocampus is involved in the 24-hour consolidation period between the introduction and recollection of novel objects [41], and this hippocampal-dependent memory assay is low stress and exposes cognitive impairments similar to those found in AD.

**Fig 1:**
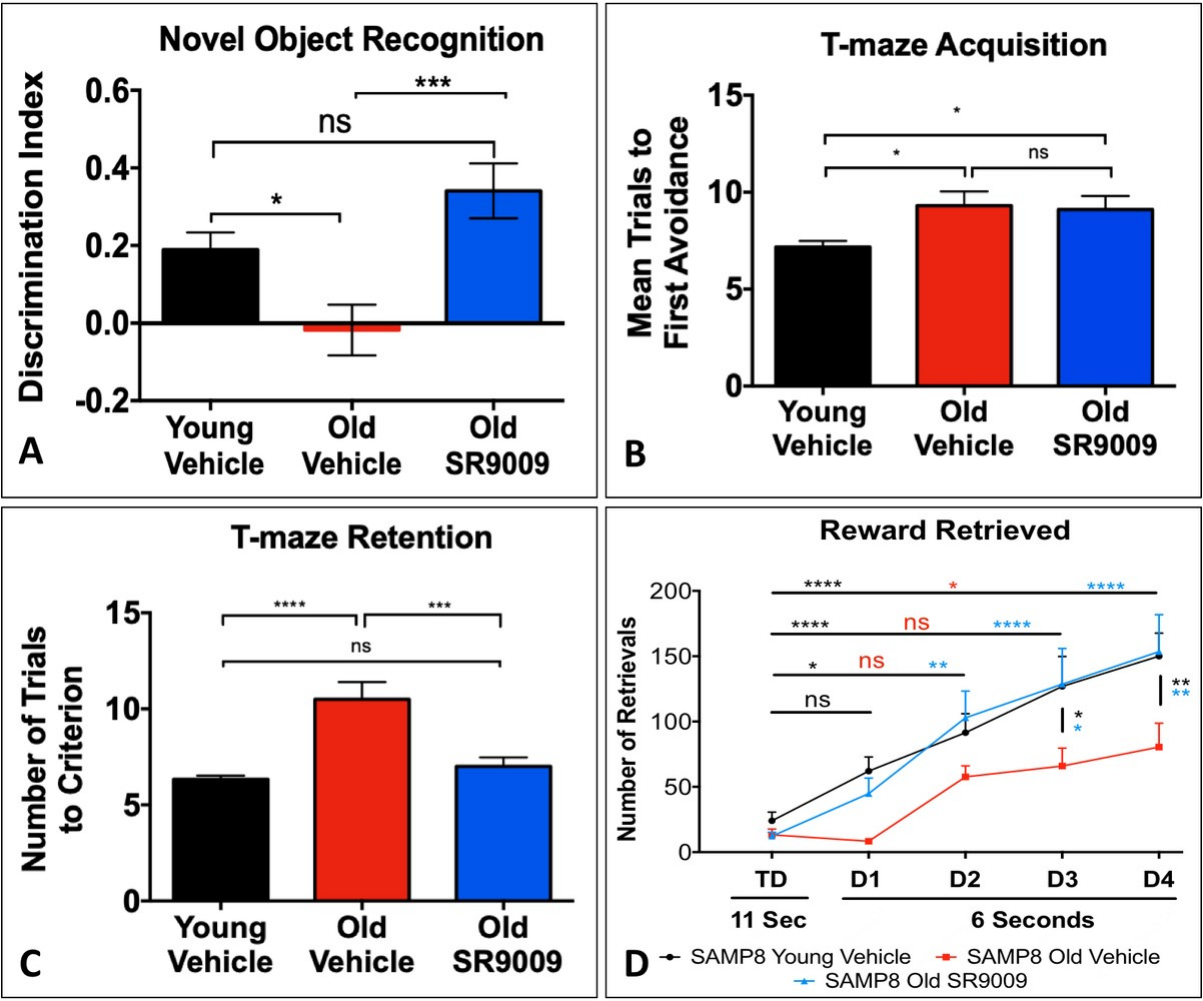
Reversal of cognitive deficits by SR9009. In novel object recognition, O-SR9009 mice had better recall of the old object and spent more time with the new **(A).** DI = (T_new object_−T_old object_) / (T_new object_ + T_old object_) (N = 11–17). In T-maze, there was no significant difference between O-SR9009 and O-V during acquisition, but neither was there significant difference between Y-V and O-SR9009 **(B)** (N = 9–12). O-SR9009 mice had improved retention over O-V after one week **(C)**. In the lever press task, O-SR9009 and Y-V mice retrieved more rewards over time than O-V and more rewards overall than O-V **(D)** (N = 9–12). These data together indicate rescued cognition in the SAMP8 mouse after up-regulation of Rev-Erb activity. *p<0.05 ***p<0.001 ****p<0.0001.

As expected, the O-V SAMP8 mice displayed decreased cognitive function in all the tests relative to the Y-V SAMP8 mice, and in nearly every case, SR9009 treatment resulted in significantly improved cognitive function in the O-SR9009 mice relative to the O-V mice (Fig 1). In many cases, the cognitive function of O-SR9009 mice was indistinguishable from the young mice. In the NOR test, the discrimination index was clearly reduced in the O-V group relative to the Y-V group, consistent with reduced cognitive function with aging. Most importantly, the O-SR9009 group displayed a discrimination index that was significantly greater than the O-V group and was indistinguishable from the Y-V group (Fig 1A).

In the T-maze foot shock avoidance task, SR9009 treatment significantly improved retention in the mice, but the effect in acquisition was less pronounced. During the acquisition phase, the O-V group displayed a significant increase in the number of mean trials to first avoidance relative to Y-V mice, consistent with age-dependent reduction in cognitive function. O-SR9009 mice were not significantly improved over O-V mice, but neither did they have significantly more trials to first avoidance than Y-V mice (Fig 1B). However, one-week post-acquisition (retention), while the O-V mice still displayed reduced cognitive function, the SR9009 treated old mice displayed results indistinguishable from the Y-V mice (Fig 1C).

SR9009 treatment also resulted in improved performance in a lever press reward conditioning assay. Previous studies show that SAMP8 mice are impaired in the lever press operant conditioning task [26]. The O-V mice retrieved fewer rewards than young mice (Fig 1D). SR9009 treatment of the old mice resulted in reward retrieval that was indistinguishable from the young mice and significantly greater than the old mice treated with vehicle alone (Fig 1D). Taken together, these data indicate that SR9009 treatment improves cognitive function in a mouse model of AD. No alteration in the levels of Rev-erbα or Rev-erbβ were noted in the brain with SR9009 treatment (S1 Fig). Using transmission electron microscopy, we also assessed the number of synapses in the hippocampal sections from each group of mice. We observed a significant decreased in synapses in the aged SAMP8 mice that was reversed with SR9009 treatment.

**Fig 2:**
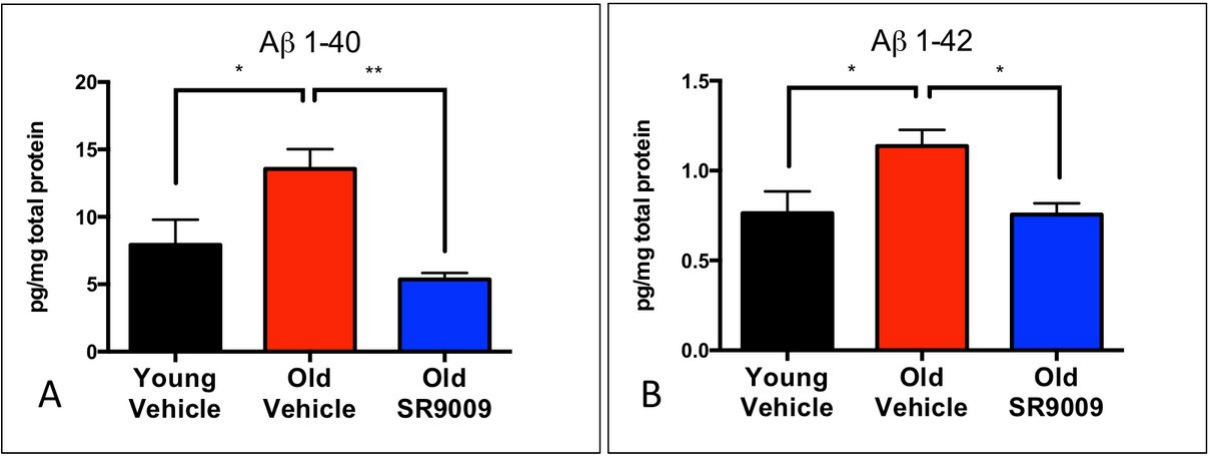
SR9009 lowers both Aβ 1–40 and Aβ 1–42 in the brain. Both species of Aβ were quantified by ELISA. Aβ 1–40 was significantly decreased **(A)**. Aβ 1–42 was significantly decreased **(B)**. *p<0.05 **p<0.01.

Aβ peptides 1–40 and 1–42 are the most recognized pathological peptides in AD [42] due to their oligomerization leading to senile plaque formation that contributes to cognitive impairment [43]. Aβ 1–42 levels are positively correlated with the age of onset for AD [44]. Our lab and others have shown amyloid accumulation in the SAMP8 mouse [29,32,33] and consistent with previous studies, we observed significantly increased Aβ 1–40 and Aβ 1–42 levels in the brains of O-V mice (Fig 2). Most importantly, we found that treatment with SR9009 was associated with significant reduction in both species of Aβ peptides (Fig 2). The levels of both Aβ 1–40 and 1–42 were actually reduced to levels indistinguishable from those observed in the young SAMP8 mice (Fig 2).

**Fig 3:**
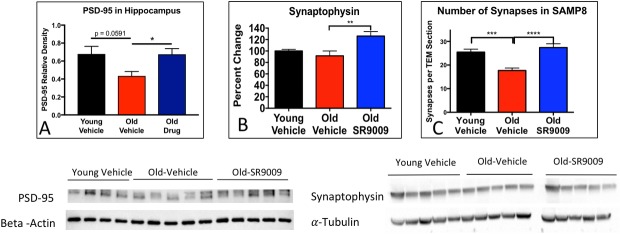
SR9009 treatment alters PSD-95 and synaptophysin expression and increases synapses. PSD-95 is a dendritic spine marker and its expression is down-regulated in the hippocampus of O-V mice. SR9009 treatment maintains levels of PSD-95 expression at levels of young mice **(A)**. The synapse marker synaptophysin is increased by SR9009 treatment **(B)**. The number of synapses identified per section in the cortex were quantified using TEM. TEM images were taken at 3000x magnification. **(C)**. N = 5 * p<0.05.

Synaptic impairment contributes to loss of cognitive function in AD [19,45]. We measured PSD-95 protein levels in the hippocampus as a marker of dendritic spine density. There was a strong trend towards reduced expression of PSD-95 in the O-V mice relative to the Y-V mice (Fig 3A), consistent with the decreased cognitive function that we observed. SR9009 treated old mice displayed increased PSD-95 protein levels relative to their vehicle treated counterparts with a level indistinguishable from the young mice (Fig 3A). Because signaling to the cortex is impaired in AD, we next investigated synaptophysin protein expression, a marker for functional synapses, in the temporal cortex of the AD mice. Interestingly, there were no differences in synaptophysin expression between the old and young vehicle treated mice, but SR9009 treatment of the old mice there was a significant increase in synaptophysin (Fig 3B). Conversely, we also assessed PSD-95 expression in the cortex and synaptophysin expression in the hippocampus. Age had no effect on expression of either of these proteins; however, SR9009 treatment did increase synaptophysin expression in the hippocampus (S2 Fig).

**Fig 4:**
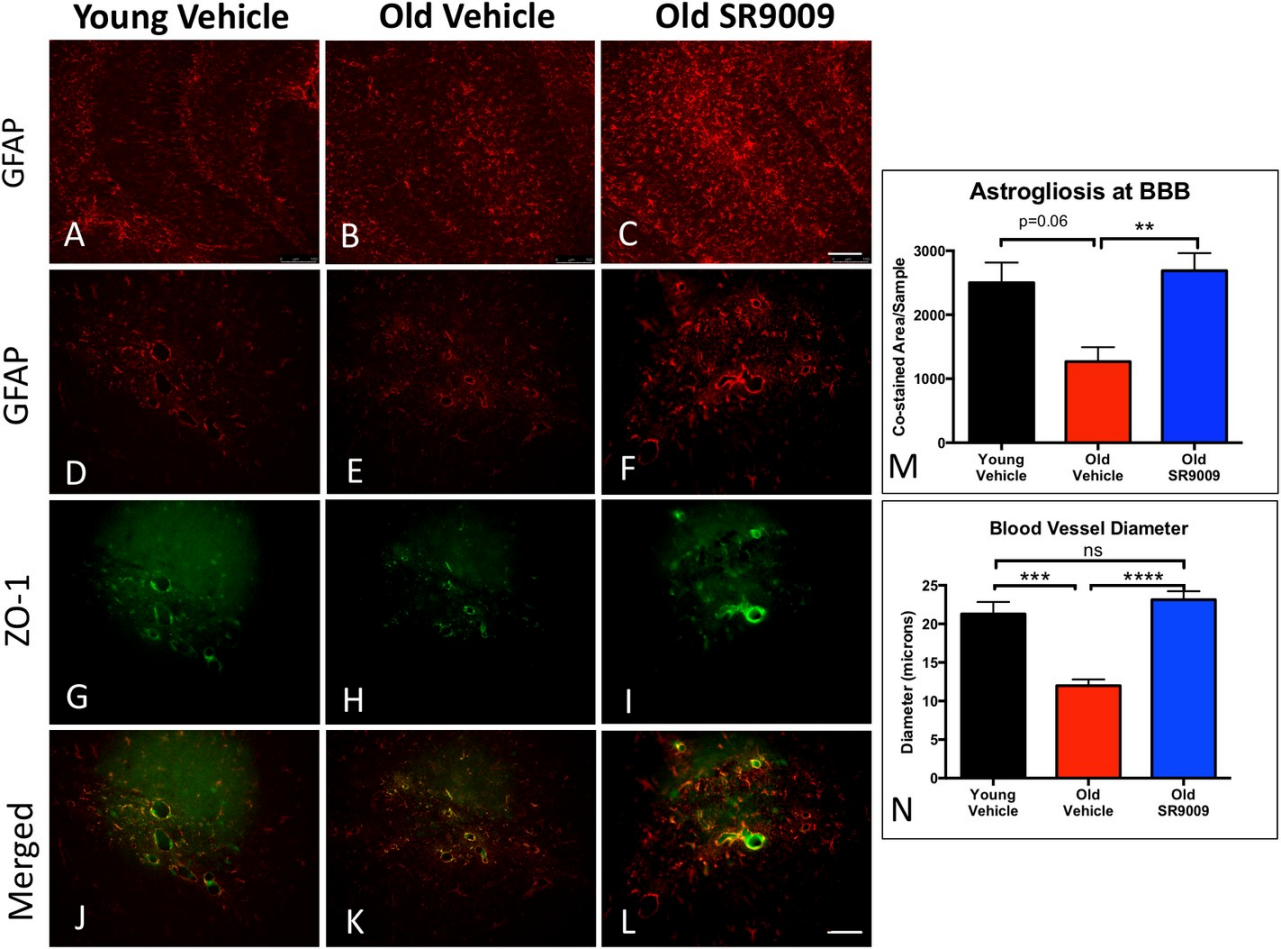
SR9009 increases astrogliosis in hippocampus, providing better support at the blood-brain barrier. IF staining for GFAP shows increase in activated astrocytes in dentate gyrus and CA3 neurons in the hippocampus of O-SR9009. There was very little GFAP staining in Y-V **(A)**. GFAP was more pronounced in O-V **(B)** and even more pronounced in O-SR9009 **(C)**. Co-staining GFAP (red) with ZO-1 (green), an endothelial marker, displayed GFAP and ZO-1 double staining in all mice **(Y-V:D, G, J O-V:E, H, K O-SR9009: F, I, L)**. Consistent with the GFAP staining alone **(A-C)**, there is more GFAP staining overall in O-SR9009 mice **(F, I, L)**. The total co-stained area was greater in O-SR9009 than O-V, but not significant between Y-V and O-SR9009 **(M)**. The diameter of blood vessels in the hippocampus were significantly greater in both Y-V and O-SR9009 than O-V, but there was no significant difference between Y-V and O-SR9009 **(N)**. Scale bar in L consistent for D-L: 50 μm. Scale bar for A-C in image, 100 μm. *p<0.05 ***p<0.001 ****p<0.0001.

Blood brain barrier (BBB) dysfunction has been shown to be present in AD and has been suggested to play a role in the pathogenesis of this disease. In order to determine if we altered BBB functionality with SR9009 treatment, we examined the expression of the astrocyte marker, GFAP, by immunofluorescence in the hippocampus. We observed an increase in GFAP positive activated astrocytes in O-SR9009 mice relative to either the young and old vehicle treated mice (Fig 4A–4C).

Functional astrocytes maintain the integrity of the BBB, so we used ZO-1 as an endothelial marker for blood vessels and co-stained with GFAP for astrocytes. Consistent with our staining for GFAP alone, we found that there was more robust staining for GFAP overall in the O-SR9009 group than in the other groups (Fig 4D–4F). Co-staining for GFAP and ZO-1 displayed a strong trend towards reduction in O-V mice relative to Y-V mice and importantly, SR9009 treatment (O-SR9009) maintained co-staining levels that were equivalent to Y-V and significantly elevated compared to O-V mice (Fig 4M). Analysis of co-stained area reveals a trend towards reduced signal in the old vehicle treated mice (Fig 4M). SR9009 treatment of the old mice led to an increase in co-stained area relative to the old mice that were vehicle-treated (Fig 4M). Vessel diameter, which is characteristically reduced in AD [46,47], was also assessed. Old vehicle treated mice displayed significantly reduced vessel diameter relative to the young mice and SR9009 treatment in the old mice substantially increased vessel size so there was no difference between Y-V and O-SR9009 (Fig 4N).

**Fig 5:**
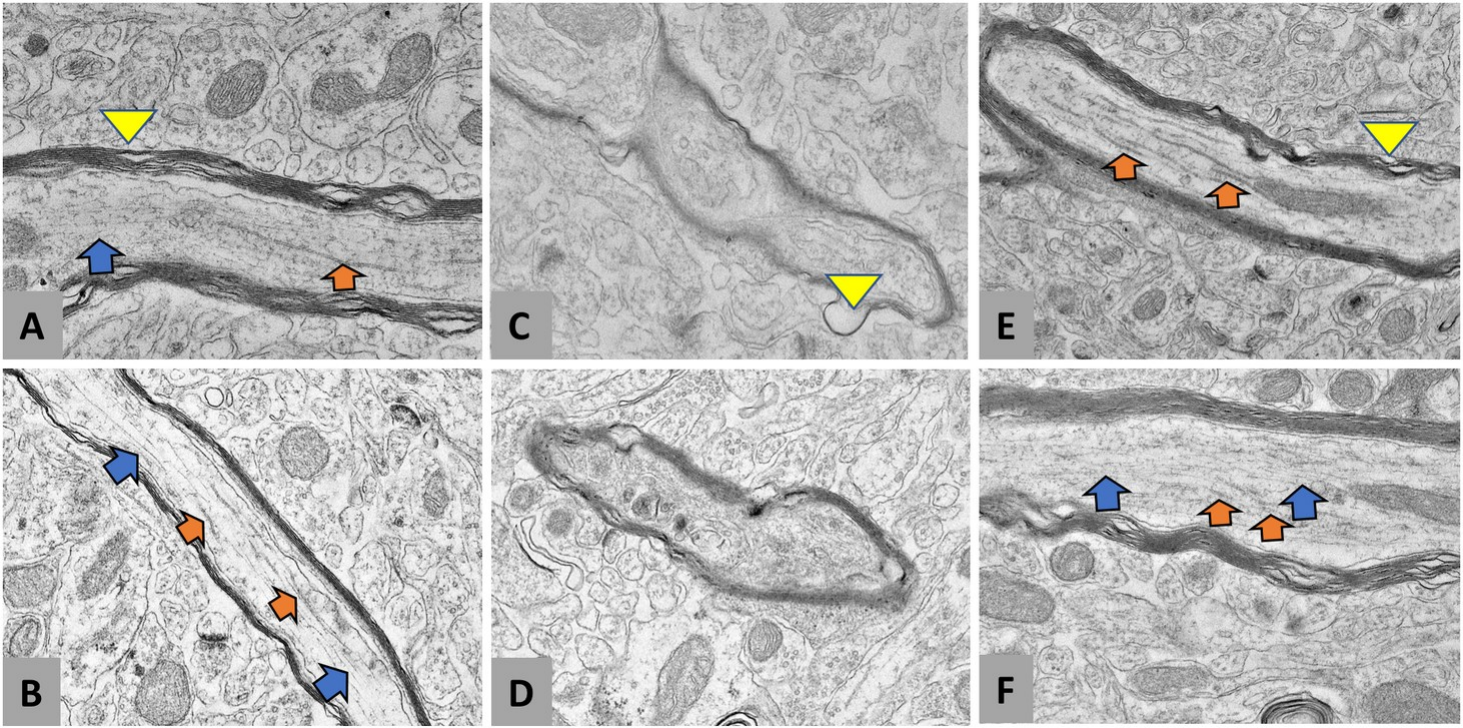
SR9009 rescues axoskeletal breakdown. Neurofilaments and microtubules supporting axonal structure in the Y-V group **(A, B)**, O-V group **(C, D)**, and O-SR9009 **(E,F)**. Blue arrows depict neurofilament bundles and orange arrows depict microtubule sections. Both are missing in the axoskeletal structure of O-V mice. Aberrant myelination (yellow arrowheads) is found in all three groups, suggesting that the pathological breakdown in myelin structure begins before the onset of memory deficits.

Axoskeletal abnormalities are also commonly observed in AD [48,49]. Using transmission electron microscopy, we readily observed abnormalities in oligodendrocyte myelination and neurofilaments in the old SAMP8 mouse. Axoskeletal aberrations arising in the old vehicle treated group that were not observed in the young or old SR9009 treated mice are shown in Fig 5. In the O-V mice, neurofilaments displayed loss of inter-axonal striatal structure, suggesting a disruption in axonal transport and a decrease in the number of healthy axons. We also observed significant deterioration of myelin structure in the old mice and interestingly, it was also noted in the young mice but to a lesser extent. O-SR9009 mice also displayed a deterioration of myelin structure that was similar to that of the Y-V mice.

*Rev-erbα* null mice have demonstrated cognitive dysfunction characterized by deficits in working and short-term memory (Y-maze) and long-term memory (NOR and contextual fear conditioning) [9]. Other hippocampal dependent behaviors such as nest building were also deficient [10]. Therefore, loss of REV-ERB function leads to cognitive deficits. Consistent with the genetic loss of function of REV-ERB causing reduced cognitive function, in the current study, we found that pharmacological gain of function of REV-ERB was sufficient to reverse multiple cognitive deficits in the SAMP8 mouse model of sporadic AD. The small molecule REV-ERB agonist, SR9009, increases the transcriptional repressor activity of this nuclear receptor, allowing us to study gain-of-function. Aged SAMP8 mice treated with SR9009 performed better than aged SAMP8 mice treated with vehicle in NOR, T-maze foot shock avoidance, and lever press operant conditioning assessments. It is clear that activation of REV-ERB led to an improvement in the performance of hippocampal-dependent memory tasks.

The long term NOR assay (24 hours) is a hippocampus-dependent task, as is the T-maze foot shock avoidance assay [41]. The hippocampus is responsible for consolidating the recognition of the training object or avoidance of the foot shock into long-term memory. During acquisition for the T-maze task, while the SR9009 treated mice did not have significantly improved acquisition over the old vehicle treated mice, they did have improved retention of the task. This further suggests that hippocampal function is being rescued in the SAMP8 mouse model when treated with SR9009, as the hippocampus is still consolidating the task to long term memory.

Learning and memory also integrates multiple systems of memory, implicating the connectivity between hippocampus and striatal nuclei in cognitive tasks [50]. Here, we show that SR9009 improves the functional integration of multiple structures in the brain. While the mechanism is still unclear, we see in the old vehicle group that, even within the group, learning the task to retrieve a reward even compared to the previous test days does not reach a significant increase in reward retrieval until the final day of the test. This would suggest that, in the SAMP8 mouse, there is a breakdown in the integration of signals from the hippocampus and the limbic system, and that treatment with SR9009 reverses that breakdown.

Accumulation of Aβ peptides leads eventually to AD senile plaques. Prior to this pathological stage, amyloid precursor protein (APP) is processed at neuronal synapses, and Aβ oligomers are released into the extracellular matrix to have physiological effects [51]. Aβ in minimal amounts physiologically benefits memory, but excessive concentrations cause pathological effects [52]. We observed a clear reduction in Aβ 1–40 and 1–42 levels in mice treated with SR9009 with levels reaching those that were indistinguishable from the young mice. The mechanism underlying REV-ERB’s reduction of Aβ levels is unclear, but the reduction is consistent with the improvement of cognitive function in the SR9009 treated mice. The decreased Aβ in the O-SR9009 group, in combination with improved cognition, suggests that increased Aβ levels are relevant in the SAMP8 model of AD and the levels are inversely correlated with cognitive function. Furthermore, Aβ toxicity is responsible for restructuring synapses in the brain [53,54], therefore decreased Aβ may be affecting synaptic plasticity in the SR9009-treated mice.

Diminishing synapses are one of the primary culprits in early loss of cognitive ability in the pathological progression of AD [55]. Synapse loss has long been documented in early and late forms of AD [45,56], and dendritic spine density correlates with improved learning and memory [57]. The increase in the pre- and post-synaptic markers PSD-95 and synaptophysin suggests that SR9009 alters cellular pathways leading to synaptic pruning in SAMP8 mice, thereby reversing the synaptic deficits in this AD model and leading to improved cognition.

Alongside the increase in synaptic proteins, we observe increased astrogliosis in the O-SR9009 group. This suggests that increased REV-ERB function improves cognition through positive glial activity. Astrocytes are known to be involved in synapse support as well as regulation of blood flow [58,59]. In the past, our lab has shown impaired blood brain barrier and Aβ efflux in SAMP8 mice [29,30,33]. Increased blood vessel diameter in the SR9009-treated mice suggests increased volume of blood flow throughout the brain. Multiple studies have shown that decrease in blood volume through the brain is common in AD and other dementias [60,61]. Pharmacological activation of REV-ERB appears to reverse the effects of decreased blood flow, both by increasing blood vessel diameter and through the increased astrocyte activity which supports blood vessels.

Typically, astrogliosis is a marker of neuronal injury. However, recent research suggests that upregulation of astrocytes is not always detrimental, but may be helpful in many circumstances, and the phenotype of reactive astrocytes will vary with type of injury [62–64]. As both Y-V and O-SR9009 animals retained good cognitive function, this suggests that the astrogliosis we observe in the old SR9009-treated SAMP8 mice is not detrimental to the animal. Functional astrocytes provide a support network for the high energy demand of the neural cells [59], thus it is possible that the astrogliosis that we observe is merely correlating to an increase in support for more active neurons in the SR9009 treated mice. This suggests that, as a negative regulator of gene expression, REV-ERB may be suppressing the toxic activity of reactive astrocytes, allowing for the neurotrophic phenotype to play a more central role. Of course, the most important endpoint for assessing potential toxic activity of the reactive astrocytes is that the cognitive function of SR9009-treated animals, which illustrate that any negative impact of astrogliosis is outweighed by the positive impact.

Breakdown in neurofilament structure exemplified an axonal abnormality unique to the O-V group. Neurofibrillary tangles are a hallmark of AD caused by hyperphosphorylated tau. However, it has been suggested that breakdown in microtubule structure precedes the hyperphosphorylation of tau [65]. The disrupted structures that appear in the axons of O-V SAMP8 mice support this, as there was no change in hyperphosphorylation of tau between groups (S3 Fig). The O-SR9009 mice show no signs of the abnormal neurofibrillary structure, suggesting that gain of REV-ERB function reverses the downstream effect that causes the breakdown in axoskeletal structure. The axon carrying the signal must not only remain energetically efficient, but also maintain structural integrity in order for efficient neurotransmission. In the SAMP8, it appears that healthy axon structure contributes significantly to cognitive health. Breakdown in neurofilaments and microtubules in the axons of the 12-month-old mice damages cognition in the SAMP8. To our knowledge, this is the first work to show that the axons in a 12-month-old SAMP8 mouse form these tangle-like structures.

In human studies, patients with progressive dementia exhibit a loss of neurofilament heavy and light chain proteins as compared to their age-matched non-demented counterparts [66], which is similar to what is observed in the SAMP8 mouse model. Thus, the protective effect of SR9009 on degeneration of axonal structure in the SAMP8 mice may be relevant in human neurodegeneration.

Aberrant myelination has also been well-documented in AD [67,68]. In our study, we found that myelination erosion began early in life in the SAMP8 mouse but had not progressed sufficiently to cause cognitive impairment at 4 months of age. However, the breakdown in myelination was particularly apparent at 12 months of age. It is as yet unclear if this is due to a decrease in oligodendrocytes or a deficiency in the myelination process. However, by 12 months of age, the neuronal signaling was impaired to the point of cognitive impairment, but SR9009 was able to rescue the effects of myelination breakdown in the old mice. However, this is consistent with previous studies that show that, in neurodegenerative diseases such as AD, the deleterious effects on the brain begin immediately after adolescence[69]. Therefore, it is not surprising that the 4-month-old SAMP8 mice do not yet display the cognitive deficits of the 12-month-old control group while still displaying the abnormal myelination.

The endogenous ligand for both REV-ERBα and REV-ERBβ is heme [70,71]. Although the physiological relevance of heme as an endogenous ligand is unclear, understanding of ligand regulation of REV-ERB has allowed development of synthetic ligands that have been important for understanding the relevance of the REV-ERBs as potential drug targets. The REV-ERBs are widely expressed, and heme is produced in all cells as a critical component (prosthetic group) of enzymes of cellular energetics, among other key roles. Previously, we demonstrated a role for variations in intracellular heme levels with the physiological role of REV-ERBs in adipogenesis [72]; however, the role of the endogenous REV-ERB ligand in terms of neuronal function and cognition is not clear. What is clear is that heme metabolism appears to be highly dysregulated in AD. Heme oxygenase 1 (HO-1), the key heme degrading enzyme induced by both heme and oxidative stress, is substantially upregulated in the brains of patients with AD. This is consistent with the supposition that AD pathology is associated with significant oxidative stress [73–75]. The typical physiological response to decreased heme levels is to increase the expression of the rate-limiting heme synthesis enzyme aminolevulinate synthase 1 (ALAS1), but in the case of AD the level of expression of ALAS1 was decreased by ~90% in the brains of patients [74]. Of course, increased expression of HO-1 (heme degradation) and decreased expression of ALAS1 (heme synthesis) would lead to a situation where dysregulated heme levels could cause significant cellular stress and, from the perspective of REV-ERB, normal physiological activity would be decreased. Loss of REV-ERBα function is known to be associated with reduced cognitive function [9–11] and lower levels of heme would translate into less active REV-ERBα and REV-ERBβ, thus reducing cognitive function. Currently, we do not know if loss of REV-ERB function leads to AD-like pathology beyond cognitive dysfunction. SR9009 treatment may activate REV-ERB to levels that are more aligned with physiological function if heme levels were normal, which may have a beneficial effect on neuronal function.

## Conclusions

We have demonstrated that gain of REV-ERB function through pharmacological activation with SR9009 reversed cognitive defects in an AD mouse model. Mechanistically, we noted that SR9009 led to decreased Aβ levels in the brain as well as increased expression of markers of synaptic health and improved axoskeletal structure. These data clearly suggest that targeting of REV-ERB may be a method to effectively treat AD as well as other cognitive disorders.

## Supporting information

S1 Fig Rev-erb expression is not altered by SR9009 treatment. Cortex was rapidly dissected and flash frozen in liquid nitrogen for gene expression analysis. RNA was extracted using TRIzol extraction techniques, and gene expression of *Rev-erbα* and *β* was analyzed using Real Time qPCR. There were no changes in REV-ERB-α or REV-ERB-β gene expression either in the old SAMP8 mice or those treated with SR9009.
(TIF)

S2 Fig Expression of PSD-95 (Cortex) and synaptophysin (Hippocampus). PSD-95 and synaptophysin expression were assessed by western blot. Young (4-month old) or Old (12-month old) SAMP8 mice treated with vehicle were compared to old SAMP8 mice treated with SR9009 for 4 weeks.
(TIF)

S3 Fig p-Tau T231 levels are not altered by SR9009 treatment. Western blot assessment of p-Tau T321 revealed no differences between Y-V, O-V and O-SR9009 treated groups.
(TIF)
