# Supplementary figures and images for: Pharmacological activation of the nuclear receptor REV-ERB reverses cognitive deficits and reduces amyloid-β burden in a mouse model of Alzheimer’s disease
Source: PLoS One. 2019 Apr 11;14(4):e0215004. doi: 10.1371/journal.pone.0215004 (PMC6459530; doi:10.1371/journal.pone.0215004)

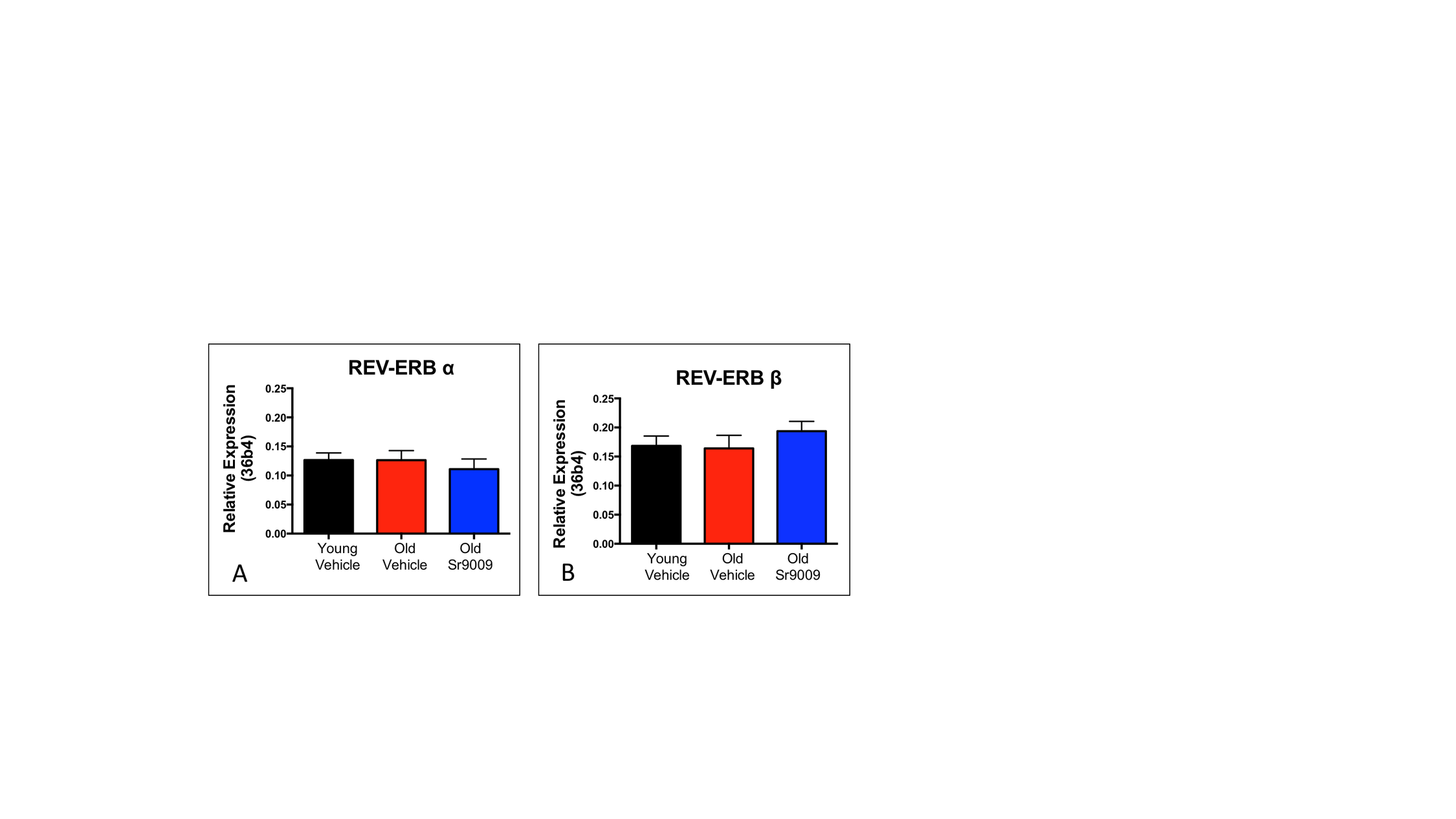

Supplement: S1 Fig — Cortex was rapidly dissected and flash frozen in liquid nitrogen for gene expression analysis. RNA was extracted using TRIzol extraction techniques, and gene expression of Rev-erbα and β was analyzed using Real Time qPCR. There were no changes in REV-ERB-α or REV-ERB-β gene expression either in the old SAMP8 mice or those treated with SR9009. (TIF) [file pone.0215004.s001.tif]

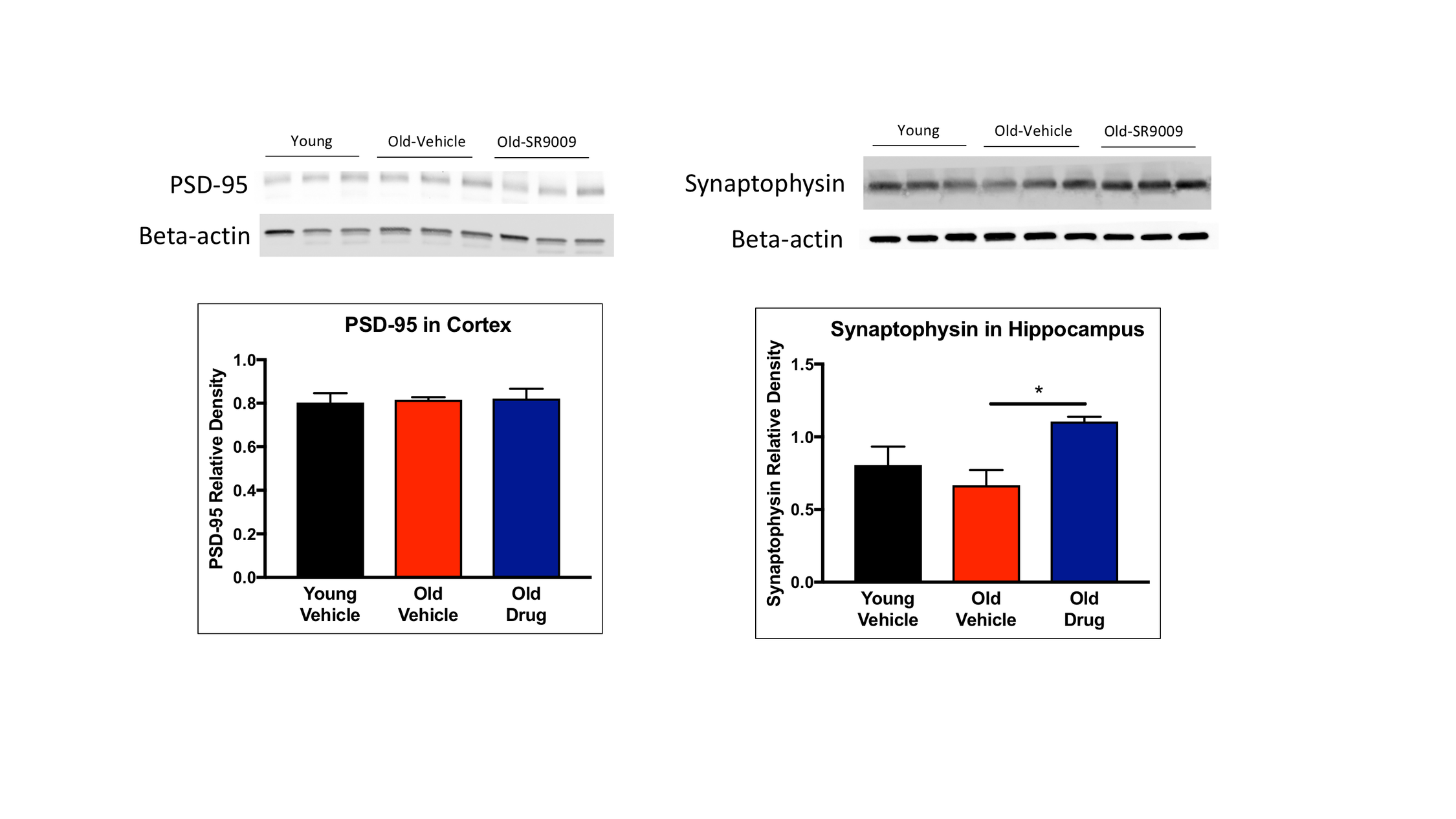

Supplement: S2 Fig — PSD-95 and synaptophysin expression were assessed by western blot. Young (4-month old) or Old (12-month old) SAMP8 mice treated with vehicle were compared to old SAMP8 mice treated with SR9009 for 4 weeks. (TIF) [file pone.0215004.s002.tif]

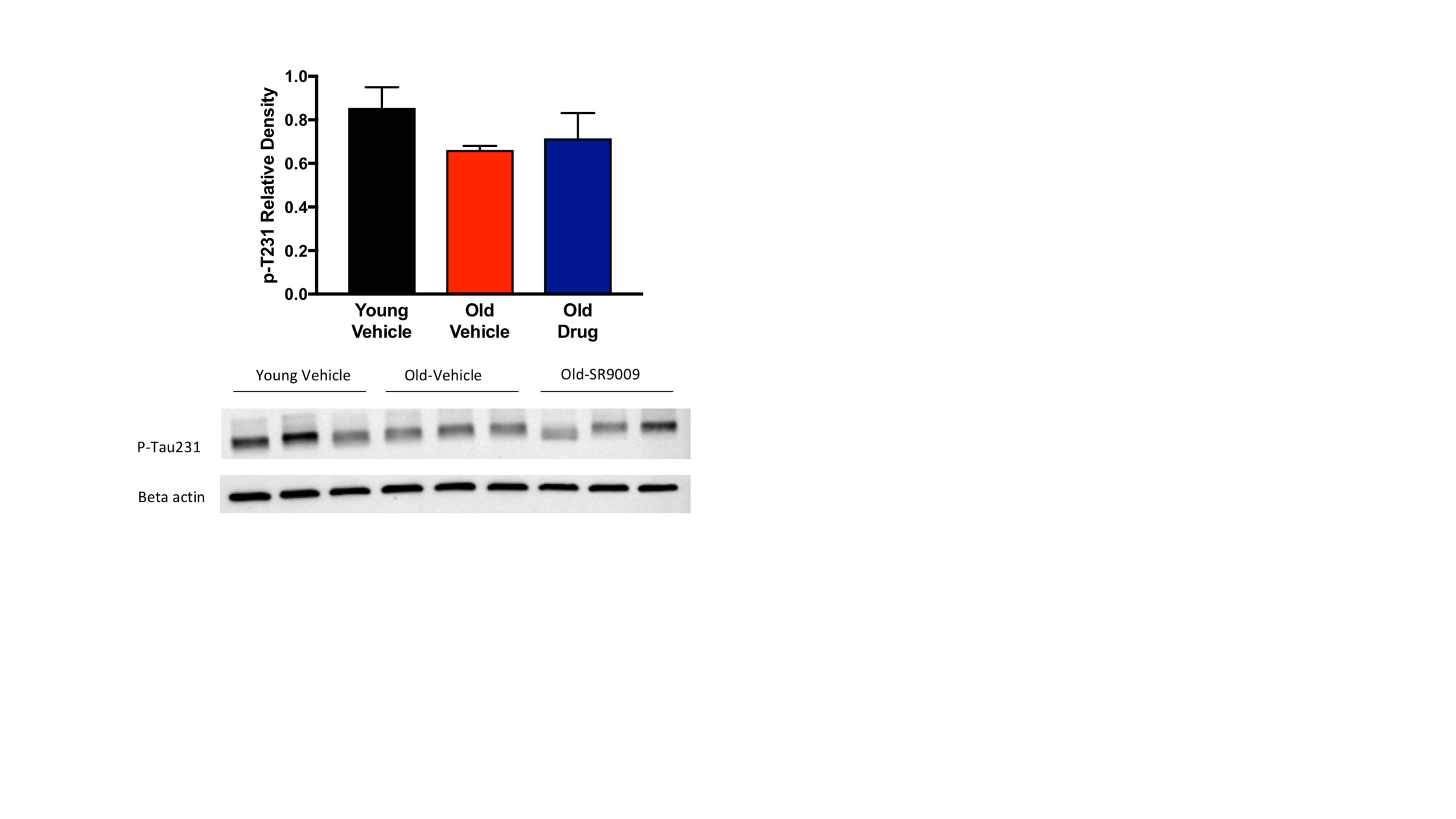

Supplement: S3 Fig — Western blot assessment of p-Tau T321 revealed no differences between Y-V, O-V and O-SR9009 treated groups. (TIF) [file pone.0215004.s003.tif]
